# Attitudes and decision-making processes of Midwakh smoking among adult males in UAE

**DOI:** 10.1192/bjo.2021.624

**Published:** 2021-06-18

**Authors:** Hamid Alhaj, Rahaf Abughosh, Batool Aldaher, Asma Elhewairis, Ahmed Ali, Fatima Alnaqbi, Shaikha Alzaabi, Basema Saddik

**Affiliations:** 1 Sheffield University, University of Sharjah; 2 University of Sharjah

## Abstract

**Aims:**

Midwakh, which involves smoking an Arabian tobacco blend typically mixed with herbs and spices, has recently become a major health concern due to a spreading popularity among adolescents and young adults in the United Arab Emirates (UAE). It is known to contain a higher nicotine content than cigarettes, potentially increasing the risk of addiction, despite contrary popular belief among young smokers. Yet, little is known about attitudes and decision-making processes involving this emerging smoking behaviour. The aim of this study was to ascertain the knowledge, attitudes and practices of Midwakh use among adult males in the UAE.

**Method:**

A cross sectional study was conducted among male adults in Abu Dhabi, Dubai and Sharjah. A total of 500 participants completed self-administered validated questionnaires, which consisted of 30 questions that targeted the public's understanding, perception and use of Midwakh. Data were analysed using SPSS 23. Percentages and means were calculated for demographic data and Chi-Square was utilised to measure relations between categorical variables. Odds Ratio (OR) was used to estimate how strongly a predictor was associated to an outcome. A p-value less than 0.05 was considered statistically significant.

**Result:**

The prevalence of smoking Midwakh was 34.8% among the study sample. Males between ages 26 to 35 were found to be 4.48 times (95% CI: 1.59–12.66) more likely to be current Midwakh smokers than any other age groups (P = 0.01). Emiratis in the study were 5.92 times (95% CI: 2.83–12.35) more likely to smoke Midwakh than expats. 65% of respondents reported willingness to smoke Midwakh if it was offered to them. Adults with 3-4 close friends who smoke Midwakh were 6.8 times (95% CI: 2.08–22.41) more likely to smoke Midwakh themselves. Knowledge of being unsafe was cited in 62% of the participants as a cause of quitting Midwakh within two years.

**Conclusion:**

Our results demonstrate a significant impact of peer pressure on the decision-making process of Midwakh smoking. The high prevalence among young male residents warrants a multi-agency public health approach to tackle the issue. Culturally sensitive campaigns raising awareness to the harmful effect of Midwakh including its addictiveness appear to be essential. Further research investigating the effects of a targeted Midwakh-smoking cessation approaches is warranted.

